# Effect of Contrast‐Enhanced CT Brain on Renal Function in Potential Intraarterial Therapy Patients in the Emergency Department

**DOI:** 10.1155/emmi/9010553

**Published:** 2026-08-24

**Authors:** Cheuk Ki Chan, Chi Li, Ping Chung Gordon Lee

**Affiliations:** ^1^ Department of Accident and Emergency, Caritas Medical Centre, Kowloon, Hong Kong, caritas.org.hk; ^2^ The University of Hong Kong, Pok Fu Lam, Hong Kong, hku.hk

**Keywords:** contrast-induced acute kidney injury, intraarterial therapy, stroke

## Abstract

**Introduction:**

Timely imaging is the cornerstone of hyperacute stroke management in emergency departments (EDs). However, the potential nephrotoxicity of iodinated contrast media remains a significant concern for clinicians when considering potential candidates for intraarterial therapy (IAT). This study aimed to investigate the effect of contrast‐enhanced computed tomography (CT) on renal function among potential IAT candidates in a local ED.

**Methods:**

We conducted a retrospective cohort study of 112 patients at an ED in Hong Kong who underwent contrast‐enhanced CT brain for IAT eligibility screening. Renal function was assessed via serum creatinine and estimated glomerular filtration rate (eGFR) at baseline and postprocedure. Subgroup analyses were performed to evaluate the impact of age, preexisting chronic kidney disease (CKD; eGFR < 60 mL/min/1.73 m^2^), diabetes mellitus (DM), hypertension (HT), and medications on postcontrast renal outcomes. The primary outcome was the incidence of postcontrast acute kidney injury (PC‐AKI), defined according to the Kidney Disease: Improving Global Outcomes (KDIGO) criteria.

**Results:**

The overall incidence of PC‐AKI was low at 0.89% (*n* = 1). Postcontrast analysis revealed an overall statistically significant reduction in mean serum creatinine from 104.97 to 90.26 μmol/L (*p* = 0.011), corresponding to an improvement in mean eGFR from 70.20 to 75.13 mL/min/1.73 m^2^ (*p* < 0.001). Within the CKD subgroup (*n* = 27), mean eGFR increased by 8.67 mL/min/1.73 m^2^ (95% CI 3.71, 13.63) (*p* = 0.001). Due to the low incidence, the study was underpowered to identify risk factors for PC‐AKI, and multivariable regression was not feasible.

**Conclusions:**

Contrast‐enhanced CT brain for the workup of potential IAT patients was not associated with clinically evident renal deterioration in this single‐centered retrospective cohort. Prospective studies with larger sample sizes, standardized timing for blood tests, and systematic assessment of confounders are required to confirm these findings.

## 1. Background

Stroke is a medical emergency commonly encountered in emergency department (ED). With the advance in technology, intraarterial therapy (IAT) is now commonly adopted across hospitals in Hong Kong. It offers a wider therapeutic window and higher recanalization rates for large vessel occlusions, potentially achieving better functional outcomes. A timely iodinated contrast‐enhanced CT brain plays a vital role in selecting appropriate patients for IAT. However, postcontrast acute kidney injury (PC‐AKI), defined as a sudden deterioration in renal function occurring within 48–72 h of contrast exposure, remains a significant concern. PC‐AKI is the third leading cause of hospital‐acquired acute renal failure and is associated with increased morbidity, prolonged hospitalization, and higher mortality [1]. Effective therapeutic interventions for PC‐AKI remain notably limited once the injury has occurred.

The relationship between intravenous contrast administration and renal impairment remains a subject of intense debate. While the transition to modern low‐osmolar or iso‐osmolar contrast agents has significantly reduced nephrotoxic potential compared to the older high‐osmolar agents, a theoretical risk still exists. This is particularly concerning for high‐risk cohorts, commonly referring to patients with advanced age, preexisting diabetes mellitus (DM), hypertension (HT), or baseline renal insufficiency. Historically, data regarding contrast‐induced injury were mainly derived from intraarterial administration during coronary angiography, which involved different hemodynamic conditions and contrast protocol compared to patients undergoing contrast‐enhanced CT brain [1, 2].

For potential IAT candidates presenting with acute ischemic stroke, the urgency of diagnosis often precludes the standard prophylactic measures such as extensive prehydration. This creates a complex risk‐benefit trade‐off for clinicians. While previous studies have explored contrast safety in various settings, data remain inconclusive regarding this specific vulnerable population in the ED.

Previous studies investigating PC‐AKI risks have several limitations. First, most studies included general patients who underwent contrast CT [2, 3]. This study focuses on stroke patients with potential IAT who underwent contrast CT brain in ED. Second, local data are lacking. Kocasaban et al. [4] discussed the incidence of contrast‐induced nephropathy in patients receiving intravenous thrombolysis and contrast‐enhanced imaging for acute ischemic stroke in Turkey. Two studies carried out in the United States also reported that contrast administration does not increase the risk of nephrotoxicity [5, 6]. Meanwhile, a local study for ED patients with sepsis concluded no association between contrast‐enhanced CT with increased risks of AKI [7]. Third, much existing data were derived from hospitalized patients undergoing elective procedures where controlled hydration protocols are feasible. The ED setting presents a different physiological status that is often not adequately represented. Last, there is limited evidence on the specific risks associated with individual comorbidities and medications (e.g., angiotensin‐converting enzyme inhibitor (ACEi)/angiotensin receptor blocker (ARB), metformin, and proteinuria) in the ED patients undergoing contrast CT brain.

Therefore, this study aims to evaluate the impact of intravenous contrast on renal function and clinical outcomes within this specific emergency cohort to better inform acute management strategies.

## 2. Methods

### 2.1. Study Design

This research is a retrospective cohort study conducted to analyze the electronic medical records of adult patients presenting to the ED of Caritas Medical Centre, Hong Kong, from January 2023 to September 2025. The study sought to examine the impact of contrast‐enhanced CT scans on the renal profiles of patients identified as potential candidates for IAT in an acute setting.

### 2.2. Study Population and Data Collection

**FIGURE 1:**
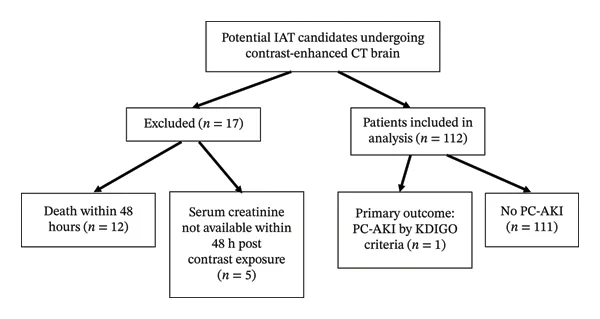
Patient selection process.

The study includes adult patients aged 18 years or older who were assessed by emergency doctors and stroke nurses as potential IAT candidates and subsequently underwent a contrast‐enhanced CT brain scan in ED. Patients were excluded if they had incomplete medical records, including missing renal function tests before or within 48 h after the imaging, patients who succumbed within 48 h of hospitalization, pregnant women, patients with unknown identity, and patients currently on renal replacement therapy were also excluded. The patient selection process is illustrated in Figure 1. Chronic kidney disease (CKD) was defined as a baseline estimated glomerular filtration rate (eGFR) < 60 mL/min/1.73 m^2^.

Data were systematically extracted from the Clinical Data Analysis and Reporting System (CDARS), the Clinical Management System (CMS), and the Electronic Patient Record (ePR). The variables collected included demographic information (age and sex), preexisting comorbidities (HT, DM, and proteinuria), and the use of specific medications such as ACE inhibitors (ACEi), ARBs, or metformin. The initial hemodynamic status defined by the presence of hypotension (systolic blood pressure < 90 mmHg) on the presentation was specifically looked at.

### 2.3. Imaging

All imaging was performed according to the standard acute stroke CT protocols in this hospital. CT images were acquired by Canon Aquilion ONE. All patients were given 80 mL normal saline at a rate of 6 mL/second, followed by injection of 100 mL contrast material (Ultravist 370 mg/mL, iopromide, nonionic water‐soluble tri‐iodinated solution) at a rate of 5 mL/second with another 160 mL normal saline given for flushing.

### 2.4. Outcome Measures

The primary outcome of this study is the change in renal function, specifically measured by comparing precontrast and postcontrast creatinine levels and eGFR, to determine if the administration of iodinated contrast is associated with a significant decline in kidney function among potential IAT candidates. The secondary outcome focuses on identifying risk factors for renal deterioration by evaluating the influence of preexisting conditions and baseline medications on the postcontrast renal outcomes.

### 2.5. Laboratory Assessment

Baseline serum creatinine was obtained prior to contrast administration. Postcontrast renal function was assessed using both the serum creatinine level within 48 h postcontrast as well as the peak available serum creatinine measurement within 7 days after contrast exposure. The individual time interval from contrast exposure to peak postcontrast creatinine sampling was not recorded and thus not reported.

### 2.6. Outcome Measures

The primary outcome of this study is the incidence of PC‐AKI, defined according to the Kidney Disease: Improving Global Outcomes (KDIGO) criteria as either an increase in serum creatinine ≥ 1.5 times baseline within 7 days or an absolute increase of 26.5 μmol/L in the serum creatinine level within 48 h of contrast exposure. The secondary outcome focuses on evaluating the change in renal function by comparing precontrast and postcontrast creatinine levels and eGFR, and on identifying potential risk factors for renal deterioration by evaluating the influence of preexisting conditions and baseline medications on the postcontrast renal outcomes.

### 2.7. Statistical Analysis

Statistical analysis was performed using SPSS. Continuous variables were expressed as means together with 95% confidence interval (CI), while categorical variables were reported as frequencies and percentages. To address the primary objective, a paired t‐test was employed to compare the creatinine and eGFR levels before and after contrast administration. For the secondary objective, an independent t‐test was performed to compare the mean change in eGFR among different subgroups to assess whether age or preexisting conditions (HT, DM, or proteinuria) or specific medications (ACEi, ARB, or metformin) significantly affect postcontrast renal function. A two‐tailed *p* value of < 0.05 was considered statistically significant.

## 3. Results

During the study period, a total of 129 patients underwent contrast‐enhanced CT brain for IAT eligibility screening. Among them, 17 were excluded due to death within 48 h of hospitalization or renal function test not performed within 48 h postcontrast. A total of 112 patients were included in this study with mean age 68.96 (95% CI 66.36, 71.57). Fifty‐seven (50.9%) of them were female. Forty‐three (38.4%) of them were 75 years old or above. Comorbidities were prevalent within the cohort, including HT (65.2%), DM (26.8%), and the use of ACEI/ARB medications (33.9%). At baseline, 24.1% of the population (*n* = 27) were classified as having CKD, defined as a preprocedural eGFR < 60 mL/min/1.73 m^2^. Demographics of patients are summarized in Table 1. Of the 112 patients included, 31 (27.7%) eventually proceeded to IAT. None of them presented with hypotension on initial assessment.

**TABLE 1:** Demographics of 112 patients who underwent contrast CT brain.

Baseline serum creatinine 104.97 (95% CI 80.95, 129.00) μmol/L		
Baseline eGFR 70.20 (95% CI 66.37, 74.02) mL/min/1.73 m^2^		
	Number	Percentage (%)
Age ≥ 75	43	38.4
Hypertension (HT)	73	65.2
Diabetes mellitus (DM)	30	26.8
Congestive heart failure (CHF)	1	0.9
Proteinuria	7	6.3
ACEI/ARB user	38	33.9
Metformin user	22	19.6
CKD (baseline eGFR < 60 mL/min/1.73 m^2^)	27	24.1

Using the KDIGO criteria defined by either an increase in serum creatinine ≥ 1.5 times baseline within 7 days or an absolute increase of serum creatinine of 26.5 μmol/L within 48 h of contrast exposure, the overall incidence of PC‐AKI was 0.89% (*n* = 1).

Paired t‐test analysis revealed a statistically significant improvement in overall renal function parameters following the administration of CT contrast. Mean serum creatinine decreased from 104.97 (95% CI 80.95, 129.00) μmol/L at baseline to 90.26 (95% CI 71.08, 109.44) μmol/L postprocedure (*p* = 0.011). Correspondingly, the mean eGFR for the entire cohort increased from 70.20 (95% CI 66.37, 74.02) to 75.13 (95% CI 71.43, 78.84) mL/min/1.73 m^2^ (*p* < 0.001). In a subgroup analysis comparing CKD patients (baseline eGFR < 60, *N* = 27) to non‐CKD patients (baseline eGFR ≥ 60, *N* = 85), both groups demonstrated significant postprocedural eGFR increases of +8.67 (95% CI 3.71, 13.63) mL/min/1.73 m^2^ and +3.75 (95% CI 1.62, 5.88) mL/min/1.73 m^2^, respectively.

Univariate analysis of potential risk factors showed no statistically significant association between the magnitude of eGFR change and the age (*p* = 0.982), baseline CKD (*p* = 0.185), the presence of DM (*p* = 0.924), HT (*p* = 0.265), or the use of metformin (*p* = 0.584) and ACEI/ARB (*p* = 0.672). The results are summarized in Table 2.

**TABLE 2:** Univariate analysis of factors associated with postcontrast eGFR change.

Subgroups	Number	Mean change of eGFR in mL/min/1.73 m^2^	95% CI	*p* value between groups
Age				
< 75	72	+3.67	+1.09, 6.25	0.982
≥ 75	40	+7.23	+4.02, 10.44	
DM				
Present	30	+3.93	−0.43, 8.29	0.924
Absent	82	+5.30	+3.01, 7.59	
HT				
Present	73	+6.37	+3.76, 8.98	0.265
Absent	39	+2.26	−0.77, 5.29	
ACEI/ARB use				
Yes	38	+4.87	+1.22, 8.52	0.672
No	74	+4.97	+2.50, 7.44	
Metformin use				
Yes	22	+4.73	+0.15, 9.31	0.584
No	90	+4.99	+2.70, 7.28	
Baseline CKD				
Present	27	+8.67	+3.71, 13.63	0.185
Absent	85	+3.75	+1.62, 5.88	

Table 3 presents a parallel subgroup analysis of the peak postcontrast serum creatinine change across the same risk factor categories. Notably, reduction in mean serum creatinine was observed across both CKD group (−40.96, 95% CI −87.96, +6.03 μmol/L) and non‐CKD group (−6.38, 95% CI ‐8.96, −3.80 μmol/L). However, with only one patient meeting KDIGO criteria for PC‐AKI, the study lacked statistical power to identify risk factors for AKI, and multivariable regression analysis was not feasible.

**TABLE 3:** Univariate analysis of factors associated with postcontrast creatinine change.

Subgroups	Number	Mean change of creatinine level	95% CI	*p* value between groups
Age				
< 75	72	−6.99	−12.22, −1.76	0.011
≥ 75	40	−28.63	−59.19, +1.94	
DM				
Present	30	−16.20	−40.39, +7.99	0.413
Absent	82	−14.17	−27.13, −1.21	
HT				
Present	73	−13.23	−23.11, −3.36	0.280
Absent	39	−17.48	−45.02, +10.04	
ACEI/ARB use				
Yes	38	−15.50	−35.37, +3.37	0.755
No	74	−14.31	−28.70, +0.08	
Metformin use				
Yes	22	−11.64	−19.09, −4.18	0.489
No	90	−15.47	−29.46, −1.47	
Baseline CKD				
Present	27	−40.96	−87.96, +6.03	< 0.001
Absent	85	−6.38	−8.96, −3.80	

## 4. Discussion

Previous studies in ED settings have shown inconsistent results in the incidence of PC‐AKI (3.2%–12%). This may be accounted by the nonspecific disease studied, differences in contrast protocol, and differences in the definitions of AKI adopted in each study [6, 8–11]. Of note, many recent studies showed no significant association between the administration of contrast agents and an increased risk of AKI [5, 8, 9, 11–14]. Our study also showed similar findings. Using the KDIGO criteria, the overall incidence of PC‐AKI was very low at 0.89% (*n* = 1). The only case meeting the criteria developed concurrent acute urinary retention which is a common complication of acute stroke. This patient subsequently had a good recovery of renal function and did not require long‐term renal replacement therapy. On the contrary, we observed an overall improvement in the mean eGFR (+4.93 mL/min/1.73 m^2^, *p* < 0.001). Overall, the risk of contrast‐enhanced CT on renal function appeared to be low in this cohort with the potential benefits of a timely reperfusion therapy. It highlighted the safety of contrast‐enhanced CT brain in this specific group of patients in ED.

We proposed that the improvement in eGFR could be explained by periprocedural hydration. In clinical practice, stroke patients are often kept nil per os and given intravenous hydration during fasting. The standard imaging protocol at our center involves the administration of 80 mL normal saline before and 160 mL after contrast injection. More aggressive hydration protocols are frequently adopted in patients with known CKD to mitigate the risk of PC‐AKI. The observed improvement in eGFR may, therefore, reflect the effect of hydration rather than the beneficial effect of the contrast media itself on eGFR.

Age, CKD, DM, and HT were traditionally considered major risk factors for PC‐AKI [15]. However, our subgroup analysis found no significant difference in the eGFR change between age below 75 versus age above 75 (*p* = 0.982), baseline CKD versus non‐CKD patients (*p* = 0.185), diabetic versus nondiabetic patients (*p* = 0.924) nor hypertensive versus nonhypertensive patients (*p* = 0.265). This aligns with Traub et al. in 2013, who found that there was a lack of accurate decision tool to identify patients at increased risk of PC‐AKI [16]. It was further demonstrated by the PRESERVE trial which concluded that even among patients who were traditionally considered high‐risk due to CKD or DM, the absolute risk of PC‐AKI was still small and was not affected by the use of prophylactic sodium bicarbonate or acetylcysteine [17].

Critically, with only one patient developing PC‐AKI by KDIGO criteria, this study was substantially underpowered to detect statistically significant associations between traditional risk factors (such as CKD, DM, or advanced age) and PC‐AKI. Multivariable regression analysis was not feasible given the extremely low event rate. Therefore, the absence of significant associations in subgroup analyses should be interpreted with cautions.

The use of metformin and ACEI prior to contrast exposure was often concerned for the increased risk of PC‐AKI. Our results showed no significant difference in renal outcomes for those on metformin (*p* = 0.584) or ACEI/ARBs (*p* = 0.672). The current American College of Radiology (ACR) guideline recommended to withhold metformin only in cases of established acute kidney injury or severe chronic impairment with eGFR < 30 mL/min/1.73 m^2^ [18]. Similarly, the controversy regarding ACEI/ARBs, which may theoretically decrease glomerular filtration pressure, has been largely settled by studies showing no increased risk of PC‐AKI in patients maintaining these therapies [19, 20].

### 4.1. Limitations of the Study

Several limitations were identified when interpreting the findings of this study. First, the very low incidence of PC‐AKI (0.89% by KDIGO criteria) severely limited the statistical power of this study to detect significant differences in subgroup analyses or to identify risk factors for AKI. Multivariable regression was not feasible due to the single event. Larger, prospective studies are needed to adequately assess risk factors.

Second, exclusion of patients who died within 48 h of hospitalization may have introduced survivor bias. Patients who died early may have been sicker overall and potentially more susceptible to renal injury. Their exclusion may have led to an underestimate of the true incidence of PC‐AKI in the broader population of acute stroke patients.

Third, the exact time interval from contrast exposure to the postcontrast blood test was not systematically recorded in this retrospective study. While we consistently used the peak available postcontrast creatinine value within 7 days postcontrast, the variable timing of blood sampling may have led to underestimation of peak postcontrast creatinine and, consequently, the true incidence of PC‐AKI. Similarly, although all patients in our cohort had their renal function tested within 48 h of postcontrast, the lack of standardized sampling time might underestimate the true incidence.

Fourth, data on several important clinical confounders were not available. Specifically, data on periprocedural hydration volumes (beyond the standardized imaging protocol saline), concurrent urinary retention, sepsis, use of diuretics, other nephrotoxic medications administered after hospitalization, NIHSS scores for categorizing stroke severity, and the use of intravenous thrombolytics were not systematically available for analysis. The absence of these data limited our power to adjust key confounders affecting renal function and represents a major limitation of this study. While we proposed the observed improvement in postcontrast eGFR is likely explained by periprocedural hydration, we could not quantify this effect.

Fifth, our cohort represented a group of patients with relatively preserved baseline renal function. Only 24.1% of them had baseline eGFR < 60 mL/min/1.73 m^2^. Patients on renal replacement therapy were excluded. This limits the generalizability of our findings to patients with more advanced renal impairment.

## 5. Conclusion

Contrast‐enhanced CT brain for the workup of potential IAT patients was not associated with clinically evident renal deterioration in this single‐centered retrospective cohort. The very low incidence of PC‐AKI (0.89% by KDIGO criteria) precluded meaningful risk factor analysis. Prospective studies with larger sample sizes, standardized timing for blood tests, and systematic assessment of confounders are required to confirm these findings.

## Funding

This research received no grant from any funding agency in the public, commercial, or not‐for‐profit sectors.

## Ethics Statement

The study was approved by the Central Institutional Review Board (IRB‐2025‐909), and the requirement for informed patient consent was waived because of the retrospective observational nature of the study.

## Conflicts of Interest

The authors declare no conflicts of interest.

## Data Availability

The research data are not shared.
